# Monitoring of Heavy Metals Content in Soil Collected from City Centre and Industrial Areas of Misurata, Libya

**DOI:** 10.1155/2013/312581

**Published:** 2013-05-24

**Authors:** M. A. Elbagermi, H. G. M. Edwards, A. I. Alajtal

**Affiliations:** ^1^Department of Chemistry, Faculty of Science, University of Misurata, P.O. Box 1338, Misurata, Libya; ^2^Raman Spectroscopy Group, University Analytical Centre, Division of Chemical and Forensic Sciences, University of Bradford, West Yorkshire, BD7 1DP, UK

## Abstract

The present paper deals with the assessment of heavy metals in soil and roadside dust around Misurata City Centre and industrial areas/roads in the period of October 2011–May 2012. The levels of Pb, Fe, Zn, Ni, Cd, Cr, and Cu in settled dust samples collected near small streets, playgrounds, gas stations and main streets in the Misurata Area have been determined by inductively coupled plasma atomic emission spectroscopy (ICP-AES). Also, the levels of same heavy metals in industrial areas have been determined. Metal concentration trend variation was also discussed in relation with traffic density and other sources of fugitive emission around different sites on each road/area. The overall mean concentration for main streets was significantly higher (*P* < 0.05) than for other small streets, where Misurata has been the centre of fierce fighting and is located in a frontline battle zone in the Libyan war; therefore most of metal concentrations in surface soil in the fighting area Tripoli Street and Benghazi Street were higher than those from the other sites (outside fighting area).

## 1. Introduction

Global industrialization and human social and agricultural activities have an effect on environmental pollution and the global ecosystem. The pollution of soil by heavy metals from automobile sources is a serious environmental issue. These metals are released during different operations of the road transport such as combustion, component wear, fluid leakage, and corrosion of metals. Lead, cadmium, copper, and zinc are the major metal pollutants of the roadside environments and are released from fuel burning, wear out of tires, leakage of oils, and corrosion of batteries, and metallic parts such as radiators [1]. Intake of heavy metals. In urban area, heavy metals in urban soil and urban road dusts can be accumulated in human body via direct inhalation, ingestion, and dermal contact absorption.

The most important sources of heavy metals in the environment are the anthropogenic activities such as mining, smelting procedures, steel and iron industry, chemical industry, traffic, and agriculture as well as domestic activities [2–12]. Chemical and metallurgical industries are the most important sources of heavy metals in soil [13–15]. Tracing an life and the environment [16]. 

The problem of environmental pollution due to toxic metals has begun to cause concern now in most of the major cities. Pollution of the environment with toxic metals has increased dramatically since the onset of the industrial revolution [17]. Soil pollution by heavy metals, such as cadmium, lead, chromium, and copper, and iron, is a problem of concern. Although heavy metals are naturally present in soil, contamination comes from local sources: mostly industry, agriculture, waste incineration, combustion of fossil fuels, and road traffic. Long-range transport of atmospheric pollutants adds to the metals in the natural environment. In recent years, it has been shown that lead levels in soil and vegetation have increased considerably due to traffic pollution, especially from the usage of leaded petrol and exhaust combustion [18].

Biomonitoring of heavy metal is still new in Libya and has its own advantages such as sample is available throughout the year, easy to identify the potential biomonitoring agent and to sample, has a wide geographical distribution, and can be relatively tolerant to pollutants [19]. 

The aim of the present study was to demonstrate the factors affecting human health through the analysis of soil samples collected from industrial areas, nonindustrial areas, and areas of heavy traffic in the City Centre of Misurata during the period of October 2011–May 2012.

## 2. Materials and Methods 

All chemicals and reagents were of analytical grade and were purchased from: (*Sigma-Aldrich, UK*). All glassware was rinsed successively with detergent and distilled water three times prior to use.

### 2.1. Data Collection and Analysis

Samples were collected during October 2011 and May 2012. 15 Soil samples (three replicates) were collected at surface level (0–10 cm in depth) from various locations to cover industrial, commercial, and residential areas. The procedure of metals determination in soil and dust samples was followed according to the reported method [20]. The soil dust samples were grounded and sieved. The sieved samples were dried at 70°C/24 hrs. One gram of soil was treated with 10 mL concentrated nitric acid heated up to dryness and then cooled. This procedure was repeated with another 10 mL concentrated nitric acid followed by 10 mL of 12 N HCl. The digested soil and dust samples were then warmed in 20 mL of 2 N HCl to re-dissolve the metal salts. Extracts were filtered using Whatman filter paper no. 40 , and the volume was then adjusted to 25 mL with 1.5% HNO_3_. Heavy metal concentrations of each fraction were inductively coupled plasma atomic emission spectroscopy (ICP-AES) assurance that was guaranteed through double determinations and use of blanks for correction of background and other sources of error. Soil pH was determined with a glass electrode with water-soil slurry (1 : 10).

## 3. Results and Discussion

Levels of heavy metals in soil and dust samples supplied from different areas of Misurata City are given in Table 1. The results of heavy metal analysis are given below with the subheadings.

The limit of detection (LOD) of the analytical method for each metal was calculated as triple the standard deviation of a series of measurements of a solution, the concentration of which is distinctly detectable above. These values were 0.001, 0.002, 0.001, 0.001, 0.003, 0.001, and 0.002 mg/kg for Pb, Fe, Cd, Zn, Cu, Cr, and Ni, respectively. Also the limit of quantification (LOQ) of the element was determined; these were 0.003, 0.003, 0.003, 0.003, 0.01, 0.003, and 0.007 mg/kg for Pb, Fe, Cd, Zn, Cu, Cr, and Ni, respectively.

**Figure 1:**
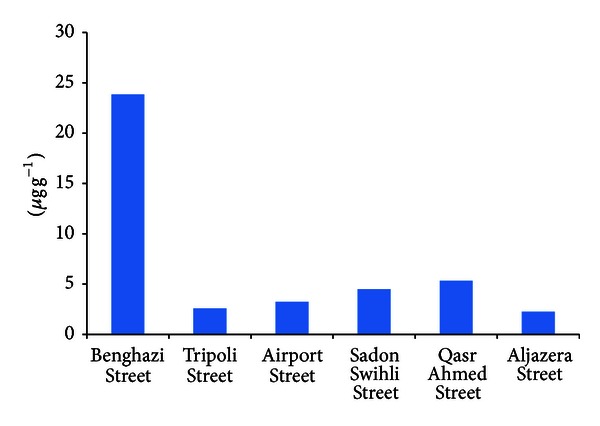
Concentration of pb, in soil.

*Lead.* In the present study, the lead content of the roadside dust ranged from 1.2 to 6.65 *μ*g g^−1^ (Table 1 and Figure 1). Maximum lead content was measured as 6.65 *μ*g g^−1^ in roadside dust of Qasr Ahmed street this area contains (Libyan Iron and Steel Company (LISCO) Port of Misurata and central petroleum station). The lead level in the Sadon Swihli Street ranges from 2.5 to 6.5 *μ*g g^−1^. The reason for the high Pb content at Taorghae Street is the heavy traffic in the area and industrial area. The lead content in the Aljazera Street was lower than in other areas.

**Figure 2:**
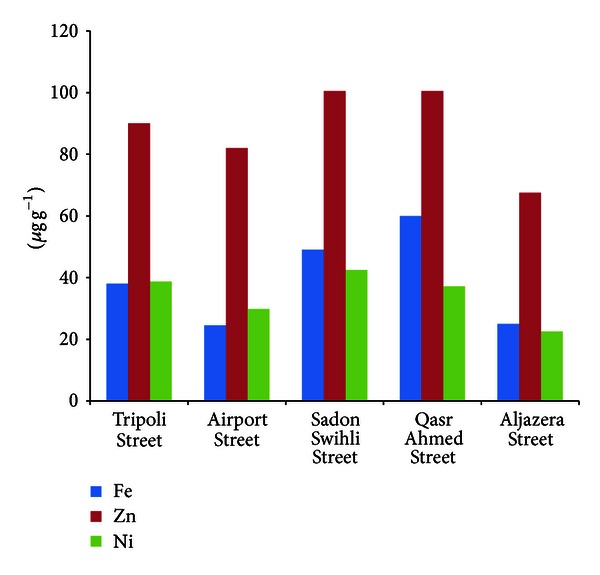
Concentration of Fe, Zn, and Ni in soil.

*Iron.* The highest Fe value as a mean was at Qasr Ahmed Street and Sadon Swihli Street samples 60 *μ*g g^−1^ and 49 *μ*g g^−1^, respectively whereas the lowest Fe concentration was at Airport Street 24.5 *μ*g g^−1^, Figure 2. 

*Zinc.* The amount of zinc in the roadside dust ranged from 42 to 146 *μ*g g^−1^ with the mean value of 94 *μ*g g^−1^. The highest Zn value was at Qasr ahmed street (146 *μ*g g^−1^), while the lowest value was at Aljazera Street (42 *μ*g g^−1^). The highest value as a mean was at Qasr ahmed street and Sadon Swihli Street samples (100.5 *μ*g g^−1^). Alloway [21] and Mcgrath and Loveland [22] reported the mean zinc concentration of 410 *μ*g g^−1^ in soil collected from the urban roadside soil in Bradford. The industrial area showed the highest mean value of 146 *μ*g g^−1^, and the seaside had the lowest concentration of 42 *μ*g g^−1^.

The mobility of the metal depends on the soil pH and also depends on the organic matter and granulometric composition of the soil. Acidic pH makes easier the solubilisation of the Zn compounds.

*Nickel.* The nickel content for the Sadon Swihli Street (36.4–45.2 *μ*g g^−1^) was the highest of all the sampling places, whereas the lowest value was at Aljazera street (13.8 *μ*g g^−1^). The concentration of nickel at Qasr Ahmed Street, Tripoli Street, and Benghazi Street were 34.5–42.8, 33.5–42.8, and 32.5–39.1 *μ*g g^−1^, respectively. Also, the mobility of the metal depends on soil pH and also depends on the organic matter and granulometric.

**Figure 3:**
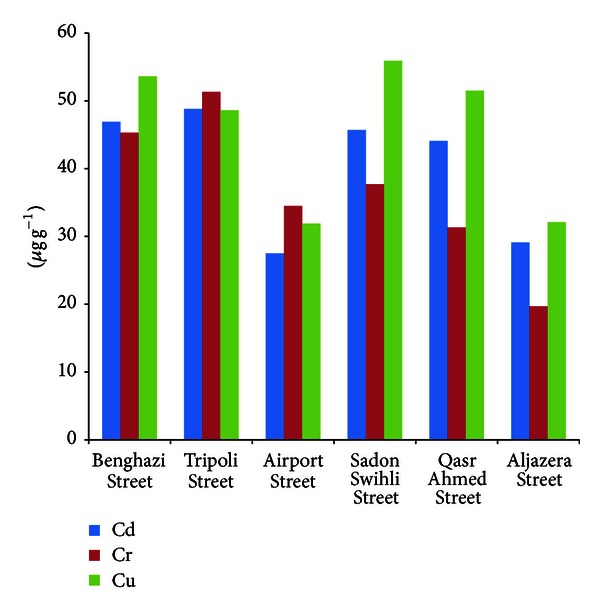
Concentration of Cd, Cr, and Cu in soil.

*Cadmium.* In the present study, the cadmium content of the roadside dust ranged from 27.5 to 48.8 *μ*g g^−1^ (Table 1 and Figure 3). Maximum cadmium content was measured as 51.2 *μ*g g^−1^ in roadside dust Tripoli street, and the lowest concentration was 12.5 8 *μ*g g^−1^ at Aljazera Street. Cadmium levels in roadside soil decrease as distancing from the main road. Also, as previously observed for Pb, the Cd levels in the heavy traffic areas (Tripoli Street, Benghazi Street, and Sadon Swihli Street) were greater than Cd levels along the residential street (Aljazera Street and Airport Street). This feature is attributed to the wear and tear of tires and the greater traffic density on the busy road compared to the residential street.

*Chromium*. The results of average Cr levels in roadside soil samples from different sites collected from Misurata Area are represented in Table 1. It is observed that the overall level of Cd lies between 45.3 and 52.4 *μ*g g^−1^ for Tripoli street, 36.7–46.8 *μ*g g^−1^ for Benghazi street, 30.4–39.8 *μ*g g^−1^ for Airport street, 33.8–43.9 *μ*g g^−1^ for Sadon swihli street, 29.7–36.8 *μ*g g^−1^ for Qasr ahmed street, and 16.7–25.9 *μ*g g^−1^ for Aljazera street. These results indicate that Cr levels among the sites of each road are significantly different. This indicates that the existence of Cr in roadside soil may be due to the tire erosion. The Cr levels in the heavy traffic areas and industrial areas were greater than Cr levels along the residential street.

*Copper*. The copper content in the roadside soil ranged from 21 to 60 *μ*g g^−1^ with the mean values of 31.9–55.9 *μ*g g^−1^ (Table 1 and Figure 3). Copper is usually present in soil within the range of 0–250 *μ*g g^−1^ [23].

Muller [24] reported the range of 1.2–1507.7 mg kg^−1^ for copper in the soil of England and Wales with a median value of 18.1 mg kg^−1^. Total copper content in most of the roadside soil was below or within the limits of the critical soil concentration of 60–125 *μ*g g^−1^ [25].

### 3.1. Contamination Levels of Heavy Metals in Soil

The contamination levels of heavy metals in urban soil, urban road dusts, and agricultural soil are assessed by using geoaccumulation index (*I*_geo_) introduced by Muller (1969). The method has been widely employed in European trace metal studies since the late 1960s [24]. The *I*_geo_ is used to assess heavy metal contamination in urban soil by comparing current and preindustrial concentrations, although it is not always easy to reach the preindustrial sediment layers. It is also employed in pollution assessment of heavy metals in urban road dust. Geoaccumulation index is computed using the following equation [24, 25]:

(1) $$\begin{array}{c} I_{\text{geo}}=\log_{2}\left(\text{Cn}/1.5\text{Bn}\right), \end{array}$$

where Cn is the measured concentration of the element in environment and Bn is the geochemical background value in soil. The constant 1.5 allows us to analyze natural fluctuations in the content of a given substance in the environment and to detect very small anthropogenic influences [26, 27]. According to Muller (1969) [24], the *I*_geo_ for each metal is calculated and classified as uncontaminated (*I*_geo_ ≤ 0); uncontaminated to moderately contaminated (0 < *I*_geo_ ≤ 1); moderately contaminated (1 < *I*_geo_ ≤ 2); moderately to heavily contaminated (2 < *I*_geo_ ≤ 3); heavily contaminated (3 < *I*_geo_ ≤ 4); heavily to extremely contaminated (4 < *I*_geo_ ≤ 5); and extremely contaminated (*I*_geo_ ≥ 5). The *I*_geo_ values for the metals in urban soil, urban road dusts, and agricultural soil for each area are presented in Table 2, respectively.

In general, Cr and Ni appear to be the least contaminated elements in all the cities, while Pb, Fe, Zn, Cu, and Cd show the highest *I*_geo_ values for most areas (Table 2). In all the areas, ranges of *I*_geo_ values for the metals are very wide. The areas of Airport street, Qasr ahmed street, and Aljazera street appear to be the least contaminated areas with low *I*_geo_ values for Cr, Cu, Pb, Zn, Ni, and Cd, while Tripoli Street, Benghazi Street, and Sadon swihli street, three heavy industrial and traffic areas, show the highest *I*_geo_ values for the metals.

The highest *I*_geo_ values for Pb (1.52) and Cd (2.23) are in Benghazi Street. The highest *I*_geo_ values for Cu (1.76) and Zn (3.56) are found in Sadon Swihli Street, while the highest *I*_geo_ value for Fe (3.43) is found in Qasr ahmed street. This indicates that the urban soil in these areas are significantly contaminated by the corresponding metals. In general, Tripoli Street and Benghazi Street have the highest *I*_geo_ values of heavy metals as they are crowded areas and were the centre of fierce fighting and are located in a frontline battle zone in the Misurata-Libyan war.

## 4. Conclusion

Heavy metal contamination in the soil from the busy roadside verges in the study area was higher as compared to the background levels for lead, iron, zinc, nickel, cadmium, chromium, and copper in residential street. These concentrations, however, were below the critical maximum levels above which toxicity is possible. The highest concentrations were detected in the samples collected from the border zone of the verges, and there was a trend of gradual decrease in the metal contents with the increasing distance from the paved roads. Also, the heavy metals concentration in industrial area was higher as compared to the nonindustrial area.

Higher Pb concentrations were found in sites with a high traffic volume on main roads and in the entrances of petrol station. It seems reasonable to conclude that Pb and Cd in roadside soil levels are significantly higher on busy roads compared to residential roads. Pb and Cd concentration levels in roadside soil decline as distancing from the main roads. We, therefore, conclude that there is a correlation between the roadside soil concentration of heavy metal and the distance from the road. It can also be observed that the frequencies with which motor vehicles stop, start, and accelerate, especially at traffic lights, may help to explain differences in the Pb levels in roadside soil. It is clear that Pb levels vary from time to time and depend on the volume of traffic.

## Figures and Tables

**Table 1:** Heavy metal concentration *μ*g g^−1^ and pH in roadside dust.

Soil location	pH	Pb	Fe	Zn	Ni	Cd	Cr	Cu
Benghazi Street	6.67	2.1–5.6	42–48	65–96	32.5–39.1	39.1–49.5	36.7–46.8	41–60
Mean		3.85	45	80.5	34.6	46.9	45.3	53.6
Tripoli Street	6.25	2.25–2.92	30–46	73–107	33.5–42.8	39.8–51.2	45.3–52.4	35–59
Mean		2.585	38	90	38.7	48.8	51.3	48.6
Airport Street	6.55	2.8–3.7	20–29	48–116	26.4–30.8	25.1–33.8	30.4–39.8	21–45
Mean		3.25	24.5	82	29.8	27.5	34.5	31.9
Sadon Swihli Street	6.89	2.5–6.5	45–53	65–136	36.4–45.2	40.1–46.7	33.8–43.9	42–66
Mean		4.5	49	100.5	42.5	45.7	37.7	55.9
Qasr Ahmed Street	6.90	4.03–6.65	48–72	55–146	34.5–42.8	38.5–47.8	29.7–36.8	38–55
Mean		5.34	60	100.5	37.2	44.1	31.3	51.5
Aljazera Street	7.30	1.2–3.3	22–28	42–93	13.8–28.4	12.5–34.8	16.7–25.9	23–41
Mean		2.25	25	67.5	22.5	29.1	19.7	32.1

**Table 2:** Geoaccumulation index of heavy metals in soil in the areas of Misurata.

Soil location	Pb	Fe	Zn	Ni	Cd	Cr	Cu
Benghazi Street	1.52	1.21	0.39	0.61	2.23	0.13	1.02
Tripoli Street	1.48	0.54	3.42	0.74	2.01	0.21	1.46
Airport Street	0.5	0.45	0.41	−0.38	0.75	−0.24	0.34
Sadon Swihli Street	1.2	2.65	3.56	0.41	0.18	0.43	1.76
Qasr Ahmed Street	0.7	3.43	3.54	−0.27	0.98	−0.8	0.32
Aljazera Street	−0.56	−0.23	0.76	−0.76	−0.35	−1.2	−0.08
